# The 2017 Ming K. Jeang award for excellence in *Cell & Bioscience*

**DOI:** 10.1186/s13578-018-0241-3

**Published:** 2018-07-24

**Authors:** Yun-Bo Shi

**Affiliations:** The National Institutes of Health, Bethesda, MD USA

## Abstract

Two research groups led by Dr. Shui Qing Ye of University of Missouri Kansas City School of Medicine, Kansas City, USA and Dr. Dihua Yu of University of Texas MD Anderson Cancer Center, Houston, USA, respectively, won the 2017 Ming K Jeang Award for Excellence in *Cell & Bioscience.*

## Editorial

We are very pleased to announce that two research groups, who each published an outstanding research article in *Cell & Bioscience* in 2017, have been selected to receive the Ming K. Jeang Award for Excellence in *Cell & Bioscience*. The Ming K. Jeang Award for Excellence in *Cell & Bioscience* was established in 2011 with a generous donation from the Ming K. Jeang Foundation to honor outstanding research articles published in *Cell & Bioscience*, the official journal of the Society of Chinese Bioscientists in America (SCBA; http://www.scbasociety.org). A committee of *Cell & Bioscience* Editors, chaired by Dr. Dong-Yan Jin, considered all research articles published in the journal in 2017 to select the following two articles to receive the award [1, 2]:

## Epigenetic regulation of Runx2 transcription and osteoblast differentiation by nicotinamide phosphoribosyltransferase

Min Ling, Peixin Huang, Shamima Islam, Daniel P. Heruth, Xuanan Li, Li Qin Zhang, Ding-You Li, Zhaohui Hu and Shui Qing Ye.

Cell & Bioscience 2017 7:27.

## 14-3-3ζ loss leads to neonatal lethality by microRNA-126 downregulation-mediated developmental defects in lung vasculature

Jun Yang, Sonali Joshi, Qingfei Wang, Ping Li, Hai Wang, Yan Xiong, Yi Xiao, Jinyang Wang, Jan Parker-Thornburg, Richard R. Behringer and Dihua Yu.

Cell & Bioscience 2017 7:58.

Congratulations to these two groups of investigators for jobs well done!

We are looking forward to receiving contributions of outstanding research articles from the scientific community in 2018 and beyond.
